# Prognostic Value of Catestatin in Severe COVID-19: An ICU-Based Study

**DOI:** 10.3390/jcm11154496

**Published:** 2022-08-02

**Authors:** Toni Kljakovic-Gaspic, Daria Tokic, Dinko Martinovic, Marko Kumric, Daniela Supe-Domic, Sanda Stojanovic Stipic, Nikola Delic, Josip Vrdoljak, Marino Vilovic, Tina Ticinovic Kurir, Josko Bozic

**Affiliations:** 1 Department of Anesthesiology and Intensive Care, University Hospital of Split, 21000 Split, Croatia; tkljakgas@kbsplit.hr (T.K.-G.); dtokic@kbsplit.hr (D.T.); sastojanovic@kbsplit.hr (S.S.S.); ndelic@kbsplit.hr (N.D.); 2 Department of Maxillofacial Surgery, University Hospital of Split, 21000 Split, Croatia; dmartinovic@kbsplit.hr; 3 Department of Pathophysiology, University of Split School of Medicine, 21000 Split, Croatia; marko.kumric@mefst.hr (M.K.); josip.vrdoljak@mefst.hr (J.V.); marino.vilovic@mefst.hr (M.V.); tticinov@mefst.hr (T.T.K.); 4 Department of Medical Laboratory Diagnostics, University Hospital of Split, 21000 Split, Croatia; dsupe@kbsplit.hr; 5 Department of Health Studies, University of Split, 21000 Split, Croatia; 6 Department of Anesthesiology and Intensive Care, University of Split School of Medicine, 21000 Split, Croatia

**Keywords:** SARS-CoV-2, COVID-19, catestatin, GDF-15, vitamin D, intensive care unit

## Abstract

Catestatin is a pleiotropic peptide with a wide range of immunomodulatory effects. Considering that patients with a severe COVID-19 infection have a major immunological dysregulation, the aim of this study was to evaluate catestatin levels in patients with COVID-19 treated in the intensive care unit (ICU) and to compare them between the fatal and non-fatal outcomes. The study included 152 patients with severe COVID-19, out of which 105 had a non-fatal outcome and 47 had a fatal outcome. Serum catestatin levels were estimated by an enzyme-linked immunosorbent assay in a commercially available diagnostic kit. The results show that catestatin levels were significantly lower in the fatal group compared to the non-fatal group (16.6 ± 7.8 vs. 23.2 ± 9.2 ng/mL; *p* < 0.001). Furthermore, there was a significant positive correlation between serum catestatin levels and vitamin D levels (r = 0.338; *p* < 0.001) while there was also a significant positive correlation between serum catestatin levels and growth differentiation factor-15 (GDF-15) levels (r = −0.345; *p* < 0.001). Furthermore, multivariate logistic regression showed that catestatin, GDF-15 and leukocyte count were significant predictors for COVID-19 survival. These findings imply that catestatin could be playing a major immunomodulatory role in the complex pathophysiology of the COVID-19 infection and that serum catestatin could also be a predictor of a poor COVID-19 outcome.

## 1. Introduction

Coronavirus disease 2019 (COVID-19) is a highly contagious infectious disease caused by the SARS-CoV-2 virus, responsible for over 3.8 million deaths worldwide. First reported in Wuhan, Hubei Province, China, in 2019, COVID-19 expanded to pandemic proportions in a short period of time, becoming the biggest world health crisis since 1918 and the influenza pandemic [1,2]. COVID-19 is an infectious disease with several pathological manifestations and pathophysiologic mechanisms where the most serious is the development of respiratory failure and consequently fatal outcome [3]. Extensive immune response by activating macrophages, monocytes and several interleukins causes damage not only in the lungs but in the whole organism [4]. Moreover, macrophages seem to increasingly produce IL-6, suggesting further development of the disease [5]. Several studies imply that the immune system plays a significant role in the disease pathogenesis [6,7,8]. In the acute phase of the disease occurs lymphopenia, which can be responsible for the virus’s escape of immune response [5] In the later stages of the disease, cells such as macrophages and neutrophils direct the immune response. These cells produce inflammatory mediators in the continuous stimulation by the virus. Hence, for a better understanding, treatment and prediction of outcomes, it is crucial to further investigate the disease and its pathophysiological mechanisms [9].

Catestatin is a pleiotropic peptide and one of the cleavage products of the pro-hormone chromogranin A. Its primary function is as an inhibitor of catecholamine secretion and stimulator of histamine release [10,11]. The principal mechanism of action is exerted through reversible and noncompetitive antagonization of neuronal nicotinic cholinergic receptors (nAChR) by occlusion of the extracellular opening of the channel pore [12]. Although a relatively novel peptide, catestatin is well investigated because of its several roles in the organism such as immunoregulation, vasodilatation, insulin resistance and antimicrobial activity [13,14,15,16]. Several studies also showed that catestatin has an important effect on the cardiovascular system [17,18,19,20]. Furthermore, a correlation between catestatin and inflammatory bowel disease was found in the study conducted by Zivkovic et al., where the level of catestatin was significantly higher in the patients with IBD than the healthy controls [21,22]. Moreover, levels of catestatin in the plasma of the patients were also elevated in other chronic inflammatory diseases, such as rheumatoid arthritis and systemic lupus erythematosus [23,24].

As aforementioned, catestatin is known for its antimicrobial activity. Several studies investigated catestatin’s potential activity against bacteria, fungi and yeast [14,25,26]. Specifically, it has been shown that catestatin’s active domain passes through the cell membrane of microorganisms and accumulates inside the cells [14]. Furthermore, a study conducted by Aung et al. showed that catestatin activates mast cell stimulation, causing migration, degranulation and release of several leukotrienes and prostaglandins [15]. Catestatin also seems to have an impact on endothelial dysfunction; a study conducted by Chen et al. showed that catestatin participates in thrombus resolution by preventing and attenuating endothelial inflammation [27]. Moreover, it has been reported that serum levels of catestatin are increased in patients with increased parameters of arterial stiffness [21]. As arterial stiffness is one of the consequences of vascular remodulation and, therefore, endothelial dysfunction, it can also be an indicator of catestatin’s impact on endothelial dysfunction. Several studies investigated endothelial dysfunction in COVID-19 patients, caused by inflammation and coagulation that affect and damage vascular cells [28]. Since one of the most severe symptoms of COVID-19 is thromboembolism and one of its hallmarks is endothelial dysfunction, it is possible that catestatin could be linked to the disease pathophysiology [29,30]. Moreover, the anti-inflammatory effects such as the reduction in monocyte migration and promotion of macrophage differentiation to the anti-inflammatory phenotype could be playing an important role in the progress of the disease [31,32,33].

Although vitamin D was initially recognized as a regulator of calcium and bone metabolism, recent research has shown that vitamin D also exhibits a profound influence on the immune system [34]. Accordingly, a body of accumulating data suggests that vitamin D deficiency is associated with poor outcomes of COVID-19 infection, which ultimately led to a widespread recommendation of vitamin D supplements to prevent COVID-19 adverse outcomes [35]. On the other hand, growth differentiation factor-15 (GDF-15), a member of the TGF-β superfamily implicated in metabolic regulation, also appears to be a strong predictor of poor outcomes in patients with a severe form of COVID-19 [36,37].

Hence, the aim of this study was to investigate the serum levels of catestatin in ICU-treated COVID-19 patients. Moreover, we wanted to compare catestatin serum levels between the patients with a fatal outcome and those who survived. The secondary goal was to evaluate possible correlations between the serum catestatin levels and anthropometric, clinical and laboratory parameters of interest. Finally, having in mind the overlapping pathophysiological pathways of catestatin and both vitamin D and GDF-15, we aimed to explore the association between serum levels among the above-noted in the setting of severe COVID-19.

## 2. Materials and Methods

### 2.1. Design and Ethical Considerations

This cross-sectional study was conducted at University Hospital of Split Respiratory Intensivist Center in the period from September 2021 to February 2022. This study was approved by the Ethics Committee at the University Hospital of Split (protocol code 500-03/21-01/14; date of approval: 29 January 2021), and it was performed according to the principles of the Declaration of Helsinki from 2013. Prior to the inclusion, all participants were informed about the purpose, course and aims of this study. Moreover, all of them signed a written informed consent form.

### 2.2. Subjects and Clinical Evaluation

The study included 152 patients treated in the intensive care unit (ICU) of the Respiratory Intensive Center. All enrolled patients were intubated and placed on mechanical ventilation on the first day of admission to the ICU. At the time of this study, the dominant variant of SARS-CoV-2 in Croatia was the delta variant (B.1.617.2). The standard references for endotracheal intubation included the following: airway protection, severe decompensate acidosis (pH < 7.2) and severe absolute hypoxemia (PaO_2_ < 50 mmHg or SpO_2_ < 90%) despite maximal noninvasive respiratory support. Included patients were treated by the up-to-date protocol in that period of time, and there were no differences in the approach of the treatment.

The inclusion criteria were: admission to the ICU after development of the respiratory failure caused by COVID-19. The exclusion criteria were: an active malignant disease; previously diagnosed autoimmune diseases; heart failure; renal failure; liver failure; vitamin D supplementation; respiratory failure due to other causes (reanimation after heart failure, CVI or polytrauma and extensive surgeries) that were treated in the ICU because of positive PCR test on SARS-CoV-2; fatal outcome due to reasons other than COVID-19.

All of the included participants underwent detailed physical examinations. Moreover, their medical history was thoroughly inspected regarding all of their personal and family anamnestic data. Clinical parameters used in the study analyses were measured on the day of the admission to the ICU. The following standard therapeutic methods and up-to-date protocols were used in patient treatment: corticosteroids, antiviral medications, anticoagulants, oxygen and other supportive therapies.

### 2.3. Laboratory Analysis

Blood samples were collected on the day of the patients’ admission to the ICU. The samples were collected according to the standard laboratory practice. All of the hematological and biochemical parameters were analyzed on the same day while the samples for catestatin and GDF-15 determination were stored at −80 C° for later analyses.

Serum catestatin levels were established by an enzyme-linked immunosorbent assay (ELISA), using a commercially available diagnostic kit (EK-053–27CE, EIA kit, Phoenix Pharmaceuticals Inc., Burlingame, CA, USA). According to the manufacturer’s instructions, the kit measurement range was 0–100 ng/mL. Reported sensitivity for catestatin was 0.05 ng/mL with a linear range of 0.05–0.92 ng/mL. Cross-reactivity with endogenous human catestatin peptide for this assay kit was 100% with the intra-assay and inter-assay coefficients of variability being <10% and <15%, respectively. The samples were not diluted before the analyses.

Serum levels of GDF-15 levels were determined using an electrochemiluminescence immunoassay on a Cobas e8000 analyzer (Elecsys, Roche Diagnostics). Reported sensitivity for GDF-15 was 400 pg/mL with a linear range of 400–20,000 pg/mL. The inter-assay coefficient of variability was 5%.

All blood samples were managed according to the international standards, in the same laboratory, by the same experienced medical biochemist who was blinded to the subjects’ group in the study.

### 2.4. Statistical Analyses

Analyses of the collected data were conducted using the statistical software MedCalc (MedCalc Software, Ostend, Belgium, version 20.110). All continuous quantitative variables were presented as mean ± standard deviation while the non-continuous variables were presented as median (interquartile range). All qualitative variables were presented as whole numbers and percentages. The normality of distribution was estimated using Kolmogorov–Smirnov test. Comparison of continuous variables between groups was conducted using Student’s *t*-test while the non-continuous variables were compared using the Mann–Whitney U test. Categorical variables were compared between groups using the chi-square test. Correlation was calculated using Pearson’s correlation for continuous variables and Spearman’s for the non-continuous variables. Additionally, independent predictors for COVID-19 survival were evaluated using the multivariate logistic regression, with OR (odds ratio), 95% CI (95% confidence interval) and *p*-value reported. The level of statistical significance was set at *p* < 0.05.

## 3. Results

There were 105 (69.0%) patients with COVID-19 in the non-fatal group and 47 (31.0%) in the fatal group. The mean age of the study sample was 76.4 ± 8.6 years. There were significantly more smokers in the non-fatal group compared to the fatal group (35 (33.3%) vs. 7 (14.9%); *p* = 0.031). There were no other significant differences between the non-fatal and the fatal group regarding the baseline characteristics (Table 1).

The non-fatal group had a significantly lower level of leukocytes (8.6 (5.8–11.2) vs. 11.8 (8.1–14.8) × 10^9^/L; *p* = 0.002), neutrophils (7.7 ± 4.8 vs. 10.1 ± 6.7 × 10^9^/L; *p* = 0.014), LDH (421.8 ± 220.8 vs. 625.1 ± 235.9 umol/L; *p* < 0.001) and hs-TnI (9.4 (6.0–14.1) vs. 18.6 (9.2–30.8) ng/L; *p* < 0.001) while the fatal group had a significantly lower level of lymphocytes (0.9 (0.6–1.3) vs. 0.7 (0.3–1.0) × 10^9^/L; *p* = 0.002) (Table 2).

**Figure 1:**
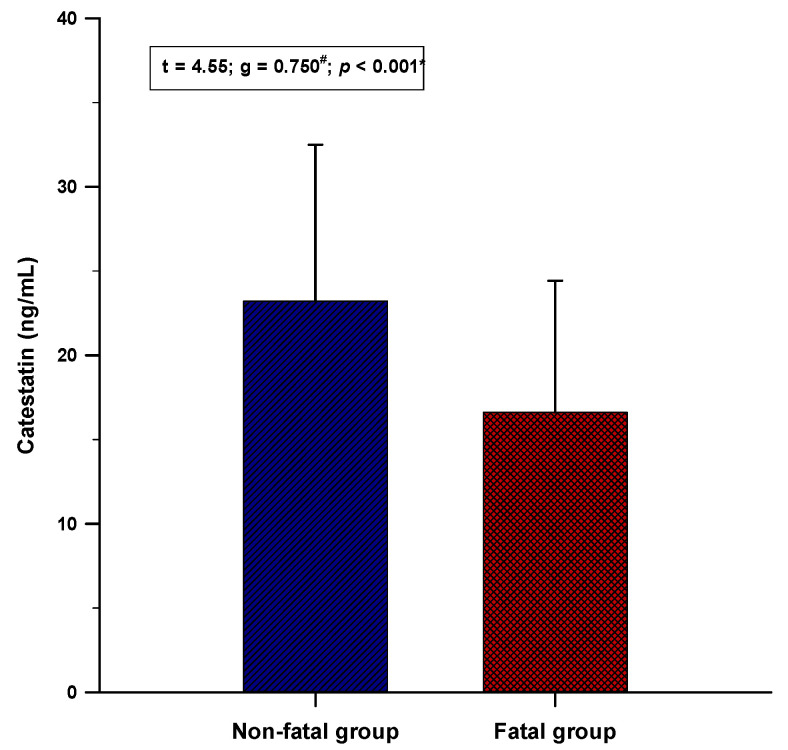
Comparison of the serum catestatin levels between the non-fatal (*n* = 105) and the fatal (*n* = 47) group. Data are presented as mean ± standard deviation * Student’s *t*-test, **^#^** Hedges’ g.

Serum catestatin levels were significantly lower in the fatal group compared to the non-fatal group (16.6 ± 7.8 vs. 23.2 ± 9.2 ng/mL; *p* < 0.001) (Figure 1).

**Figure 2:**
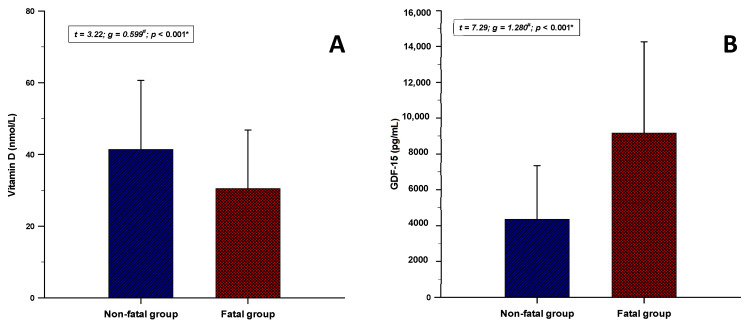
Comparison of the (**A**) vitamin D and (**B**) GDF-15 levels between the fatal (*n* = 47) and the non-fatal group (*n* = 105). Data are presented as mean ± standard deviation. Abbreviations: GDF-15—growth differentiation factor-15. * Student’s *t*-test. ^#^ Hedges’ g.

Furthermore, serum vitamin D levels were significantly lower in the fatal group (30.4 ± 16.3 vs. 41.4 ± 19.2 nmol/L; *p* < 0.001) while the GDF-15 levels were significantly higher in the non-fatal group (4354.2 ± 2989.2 vs. 9166.1 ± 5086.5 pg/mL; *p* < 0.001) (Figure 2).

**Figure 3:**
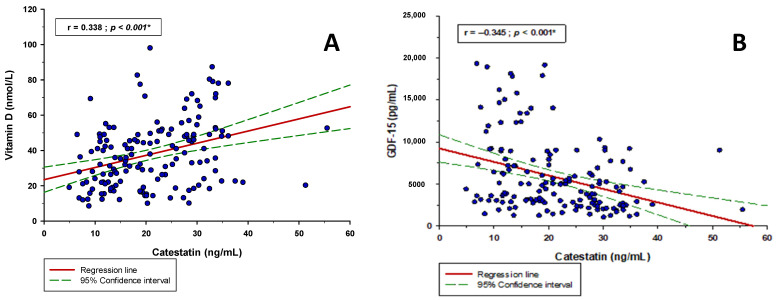
Catestatin correlation with (**A**) vitamin D and (**B**) GDF-15 in the whole study sample (*n* = 152). * Pearson’s correlation coefficient.

There was a significant positive correlation between serum catestatin levels and vitamin D levels (r = 0.338; *p* < 0.001) and significant negative correlation between serum catestatin levels and GDF-15 levels (r = −0.345; *p* < 0.001) (Figure 3). Moreover, catestatin had a significant positive correlation with systolic BP (r = 0.170; *p* = 0.036) and diastolic BP (r = 0.201; *p* = 0.013) (Table 3).

Furthermore, multivariate logistic regression showed that serum catestatin levels were a significant negative predictor (OR 0.934, 95% CI 0.886–0.984, *p* = 0.010) for a fatal COVID-19 outcome. On the other hand, serum GDF-15 levels (OR 1.037, 95% CI 1.022–1.054, *p* < 0.001) and leukocyte count (OR 1.077, 95% CI 1.003–1.157, *p* =0.008) were the significant positive predictors for a fatal COVID-19 outcome. Other computed independent variables were age, gender and vitamin D levels (Table 4).

## 4. Discussion

The results of our study show that serum catestatin levels, in the ICU-treated patients, were significantly lower in the COVID-19 fatal group compared to the non-fatal group. Moreover, there was a significant positive correlation between serum catestatin levels and serum vitamin D levels and a significant negative correlation between serum catestatin levels and serum GDF-15 levels. Additionally, the logistic regression model showed that serum catestatin is a significant negative predictor of the COVID-19 fatal outcome in our study sample. To the best of our knowledge, this is the first study that investigated serum catestatin levels in COVID-19 patients.

Previous studies have established that catestatin plays an important immunomodulatory role with an impact on macrophage differentiation and monocyte migration [31,32,38,39]. The outcomes of our study showed that serum catestatin levels were higher in both COVID-19 fatal and non-fatal ICU-treated patients when compared to the reference values of the age- and gender-matched healthy controls in earlier studies [11,17,21,24,40,41,42]. However, even though higher than normal, catestatin was still significantly lower in the fatal group than it was in the non-fatal group. While this seems paradoxical due to the fact that catestatin is usually higher in inflammatory states, some of the previously conducted studies also showed that serum catestatin levels are lower in certain diseases [16,17,31,43,44]. It is well established that catestatin diminishes the expression of integrin ligands on the endothelial cells [32,33,44]. Furthermore, its effect shows a reduction in monocyte migration while macrophages are steered toward an anti-inflammatory phenotype which consequently leads to an upregulation of anti-inflammatory markers and an increased production of IL-10 and IL-4 [31,45]. Additionally, it was shown that the subsequent expression of pro-inflammatory cytokines is severely reduced [31,33]. It is also important to address that emerging evidence suggests that SARS-CoV-2-mediated endothelial injury is an important effector of the virus, including even an overlap with ischemic-reperfusion injury [46,47]. On the other hand, it has been shown that catestatin stimulates the NO-cGMP pathway whilst inhibiting the endothelin-1 receptor, thus further substantiating that change in catestatin serum levels reflects vascular endothelial injury [48].

On the other hand, one of the hallmarks of severe COVID-19 infection is cytokine release syndrome (CRS) [49]. It was determined in several studies that the levels of proinflammatory cytokines were higher in patients with COVID-19 compared to the healthy control subjects, while among the COVID-19 infected, they were significantly higher in the ICU-treated patients than in non-ICU-treated patients [50,51]. These results suggest that excessive cytokine production is associated with the severity of the COVID-19 disease. Moreover, numerous studies showed that catestatin levels are lower in DM and obesity [16,17,31,43]. These conditions are regarded as high-risk comorbidities for a severe COVID-19 infection. It is possible that the catestatin could be one of the links between the severe COVID-19 infection and CRS. However, this hypothesis should be addressed in future larger studies.

As aforementioned, our results show a negative correlation between serum levels of catestatin and GDF-15. Previous studies have well established that GDF-15 has a major impact on the development and progress of numerous diseases due to its metabolic regulation [52]. Moreover, recent studies conducted on COVID-19 patients found that tissue damage and hypoxia consequently respond with a significant rise in GDF-15 activity [53,54,55]. All of this evidence pointed out that GDF-15 could be a strong predictor of COVID-19 severity and poor outcomes. Furthermore, it was reported that endothelial dysfunction promotes a higher expression of GDF-15 [56]. Since endothelial dysfunction plays an important role in the pathogenesis of the COVID-19 infection given that endothelial cells constitute a direct target of SARS-CoV-2, GDF-15 is secreted from the endothelial cells as a direct result of inflammation and oxidative stress produced by the disease [36,57]. As abovementioned, catestatin through its immunomodulatory effects diminishes the expression of the integrin ligands on the endothelial cells which was proved to reduce endothelial dysfunction [27]. Hence, it seems that these two peptides probably have opposing but also possibly major effects on the inflammation produced by COVID-19. Moreover, it is possible that their interconnection in the complex pathophysiological pathways could be one of the reasons why our results show significantly lower catestatin in the fatal COVID-19 group compared to the non-fatal group. Nevertheless, our logistic regression model showed that both of them are significant predictors of the COVID-19 disease outcome.

Another important result of this study is the positive correlation between serum catestatin and vitamin D levels. Vitamin D was thoroughly investigated regarding its implicated connection with a good outcome of the COVID-19 infection [58]. It was proposed that this is due to the vitamin D ability in modulating various aspects of the innate and adaptive immune systems, as well as the antiviral effect on the enveloped viruses through the upregulation of antimicrobial peptides [59,60,61,62]. Moreover, through its anti-inflammatory actions, it attenuates the acute lung injury induced by COVID-19 and consequently reduces the disease severity [63,64]. Hence, it is possible that vitamin D deficiency contributes to the induction of CRS as well as the inadequate protection and repair of the lung epithelial cells, all of which eventually cause a high vulnerability of the lungs to fatal immunity dysregulation [65,66,67]. Since, according to previous studies, catestatin also shows a similar anti-inflammatory effect, it is possible that there is an interconnection between their effects. Furthermore, two animal studies showed that chromogranin A secretion and synthesis in the parathyroid gland are directly stimulated by the increased concentration of vitamin D [68,69]. This could implicate that vitamin D deficiency possibly downregulates chromogranin A secretion and consequently a lower level of catestatin. Nevertheless, this needs to be verified in future studies.

There are several limitations to our study. We had a relatively small sample size, and it was conducted in a single center. Moreover, its cross-sectional design prevents making any causal conclusions. Even though catestatin is well researched and we are able to compare our results with other studies, we are still missing a healthy control group. In the present study, the viral load was not assessed, and such an aspect may be relevant for CST and GDF-15 serum concentrations. Lastly, we were not able to eliminate all of the confounding effects which could have possibly interfered with the results of the study.

## 5. Conclusions

In conclusion, our study showed that patients with COVID-19 have a high level of catestatin. However, when comparing patients with the fatal outcome and those who survived, the latter have significantly higher serum catestatin levels. All of this implies that catestatin could be playing a major role in the complex pathophysiology of the COVID-19 infection. Moreover, the negative correlation between GDF-15 and our logistic regression model suggests that serum catestatin could also be a predictor of a poor COVID-19 outcome. However, all of the presented evidence should be addressed in a future larger-scale longitudinal multicentric study.

## Figures and Tables

**Table 1:** Anthropometric and anamnestic data of the study population.

Parameter	Study Sample (*n* = 152)	Non-Fatal Group (*n* = 105)	Fatal Group (*n* = 47)	Test Statistics	Effect Size	*p*
Male gender	82 (53.9)	56 (53.3)	26 (55.3)	0.003	0.001	0.959 *
Age	76.4 ± 8.6	74.6 ± 8.7	78.3 ± 8.1	1.797	0.316	0.074 ^†^
Smoking	42 (27.6)	35 (33.3)	7 (14.9)	4.637	0.376	0.031 *
DM	55 (36.2)	36 (34.3)	19 (40.4)	0.297	0.024	0.585 *
AH	74 (48.7)	47 (44.8)	27 (57.4)	1.164	0.094	0.203 *
Dyslipidemia	29 (19.1)	21 (20.0)	8 (17.0)	0.044	0.003	0.834 *
CVD	39 (25.7)	23 (21.9)	16 (34.0)	1.912	0.155	0.166 *
Fully vaccinated	27 (17.8)	19 (18)	8 (17)	0.026	0.013	0.873
Total hospitalization duration	18 (14–26)	15 (12–22)	25 (17–32)	5.653	0.458	0.001 ^‡^
ICU duration	14.7 ± 7.6	12.4 ± 7.3	21.1 ± 8.1	6.562	1.151	0.001 ^†^

All data are presented as whole number (percentage), mean ± standard deviation or median (IQR). * chi-square test; ^†^ *t*-test for independent samples; ^‡^ Mann–Whitney U test; Abbreviations: BMI—body mass index; DM—diabetes mellitus; AH—arterial hypertension; CVD—cardiovascular disease; ICU—intensive care unit.

**Table 2:** Comparison of clinical and laboratory parameters between the fatal and the non-fatal group.

Parameter	Study Sample (*n* = 152)	Non-Fatal Group (*n* = 105)	Fatal Group (*n* = 47)	Test Statistics	Effect Size	*p*
Leukocytes (×10^9^/L)	9.3 (6.1–12.3)	8.6 (5.8–11.2)	11.8 (8.1–14.8)	2.998	0.243	0.002 ^‡^
Neutrophils (×10^9^/L)	8.5 ± 5.6	7.7 ± 4.8	10.1 ± 6.7	2.485	0.440	0.014 ^†^
Lymphocytes (×10^9^/L)	0.7 (0.5–1.2)	0.9 (0.6–1.3)	0.7 (0.3–1.0)	−2.891	0.234	0.002 ^‡^
Platelets (×10^9^/L)	262.2 ± 127.8	271.5 ± 127.2	241.5 ± 128.9	−1.338	0.221	0.183 ^†^
hs-CRP (mmol/L)	96.6 ± 88.0	88.9 ± 80.0	109.4 ± 93.9	1.193	0.242	0.171 ^†^
Systolic BP (mmHg)	119.7 ± 22.9	119.4 ± 21.3	120.1 ± 24.5	0.433	0.031	0.858 ^†^
Diastolic BP (mmHg)	83.0 ± 12.9	82.5 ± 12.1	83.9 ± 14.2	0.624	0.109	0.533 ^†^
D-dimers (mg/L)	2.6 ± 2.0	2.4 ± 1.9	2.9 ± 2.1	1.451	0.254	0.148 ^†^
LDH (umol/L)	520.2 ± 226.9	421.8 ± 220.8	625.1 ± 235.9	5.136	0.901	<0.001 ^†^
hs-TnI (ng/L)	12.1 (7.2–20.3)	9.4 (6.0–14.1)	18.6 (9.2–30.8)	4.943	0.400	<0.001 ^‡^

All data are presented as whole number (percentage), mean ± standard deviation or median (IQR). ^†^ *t*-test for independent samples; ^‡^ Mann–Whitney U test; Abbreviations: hs-CRP—high-sensitivity C reactive protein; BP—blood pressure; LDH—lactate dehydrogenase; hs-TnI—high-sensitivity troponin I.

**Table 3:** Catestatin correlation with clinical and laboratory parameters.

Parameter	r *	*p*
Age	−0.006 *	0.938
Total hospitalization duration	−0.009 *	0.912
ICU duration	−0.045 *	0.581
Leukocytes (×10^9^/L)	0.037 ^†^	0.652
Neutrophils (×10^9^/L)	−0.013 *	0.872
Lymphocytes (×10^9^/L)	0.112 ^†^	0.169
Platelet (×10^9^/L)	0.081 *	0.321
hs-CRP (mmol/L)	−0.032 *	0.695
Systolic BP (mmHg)	0.170 *	0.036
Diastolic BP (mmHg)	0.201 *	0.013
D-dimers (mg/L)	0.156 *	0.054
LDH (umol/L)	0.120 *	0.140
hs-TnI (ng/L)	−0.092 ^†^	0.259

* Pearson’s correlation coefficient; ^†^ Spearman’s correlation coefficient. Abbreviations: ICU—intensive care unit; hs-CRP—high-sensitivity C reactive protein; LDH—lactate dehydrogenase; hs-TnI—high-sensitivity troponin I.

**Table 4:** Univariate and multivariate logistic regression analyses of independent predictors for COVID-19 fatal outcome.

Parameter	Univariate Logistic Regression			Multivariate Logistic Regression		
	OR	95% CI	*p*	OR	95% CI	*p*
Age	1.038	0.996–1.082	0.076	1.030	0.976–1.087	0.271
Male gender *	0.923	0.462–1.842	0.820	1.172	0.497–2.761	0.716
Catestatin	0.912	0.870–0.956	<0.001	0.934	0.886–0.984	0.010
GDF-15	1.040	1.029–1.068	<0.001	1.037	1.022–1.054	<0.001
Vitamin D	0.965	0.944–0.987	0.002	1.006	0.979–1.034	0.640
Leukocyte count	1.083	1.021–1.150	0.007	1.077	1.003–1.157	0.040

Abbreviations: OR, adjusted odds ratio; 95% CI, 95% confidence interval; * Female gender was the reference group. Abbreviations: GDF-15—growth differentiation factor-15.

## Data Availability

All data sets are available on request to the corresponding author.
